# Update of students’ and lecturers’ perspectives on online learning in dental education after a five-semester experience due to the SARS-CoV-2 (COVID-19) pandemic: insights for future curriculum reform

**DOI:** 10.1186/s12909-023-04544-2

**Published:** 2023-08-08

**Authors:** Maximiliane Amelie Schlenz, Bernd Wöstmann, Norbert Krämer, Nelly Schulz-Weidner

**Affiliations:** 1 Dental Clinic - Department of Prosthodontics, Justus Liebig University, Schlangenzahl 14, 35392 Giessen, Germany; 2 Dental Clinic - Department of Pediatric Dentistry, Justus Liebig University, Schlangenzahl 14, 35392 Giessen, Germany

**Keywords:** Dentistry, Dental education, Online learning, Dental students, Dental lecturers, Questionnaires, COVID-19, Coronavirus, Curriculum development

## Abstract

**Background:**

The coronavirus disease (COVID-19) pandemic has accelerated digital transformation in dental education, resulting in a shift from face-to-face teaching to online learning. While online learning could be a common strategy in various fields, the challenge for dental education is that it depends on the requirements of clinical experience to achieve competence in performing the dental treatment. This cross-sectional study aimed to analyse students’ and lecturers’ perceptions towards online learning after five semesters of experience using a questionnaire survey.

**Methods:**

Since the spring term of 2020, the theoretical part of the curriculum has been conducted in the form of online learning using a combination of synchronous and asynchronous formats. In the following semesters, more theoretical content was shifted back from online learning to face-to-face courses. Preclinical and clinical students enrolled in the dental curriculum during the spring term 2022 semester and all lecturers with at least one year of teaching experience in face-to-face and online learning formats were asked to fill out an online questionnaire regarding the aspects of handling, didactic benefit, motivation, and overall assessment.

**Results:**

Students and lecturers rated the implementation of online learning as mostly positive, but pointed out that established ‘face-to-face’ learning could not be replaced. Moreover, the students reported personal benefits in terms of daily planning. Lecturers also benefitted as their experience increased in online teaching. For future curriculum, students demanded 49.5% (25.1) ((mean (standard deviation) of theoretical part in terms of online learning), while lecturers demanded only 34.1% (24.1).

**Conclusions:**

Despite having no prior need for online learning, students and lecturers showed a positive perspective on online learning which should be considered in the implementation and planning of future dental education. However, in terms of practical training, it cannot replace face-to-face education in dentistry.

**Supplementary Information:**

The online version contains supplementary material available at 10.1186/s12909-023-04544-2.

## Background

Until the spring term 2020, ‘distance learning’ in terms of ‘online learning’ was sporadically used for imparting dental education in most countries [1]. However, the global coronavirus disease 2019 (COVID-19) pandemic due to severe acute respiratory syndrome coronavirus 2 (SARS-CoV-2) forced lecturers at dental schools to reorganise educational format from ‘face-to-face’ teaching to online learning [2, 3]. Therefore, the COVID-19 pandemic provided the chance to accelerate digital transformation in dental education [4, 5].

While online learning could be a commonly adopted strategy in various fields, the challenge for dental education is that it depends on the requirements of clinical experience to achieve competence in performing dental treatment [6]. Patient contact is essential for dental education, especially in clinical semesters and clinical courses ensure that students master dental procedures. Skills cannot be acquired without patient contact [7]. This is also underlined by other findings indicating a lack of technical skills among students learning solely through online formats [7, 8]. This also means training in practical skills on manikins in preclinical education is mandatory [8, 9]. As a result, it is widely accepted that only theoretical learning content in dental education is suitable for digital teaching formats.

The lack of online learning opportunities cannot be ascribed to a lack of willingness towards a digital mindset among lecturers in dentistry. In the last decade, digitalisation in dental care has rapidly developed with improvements in hardware and software components [10]. Therefore, the dental curriculum has already been extended to digital dentistry, such as computer-aided design/computer-aided manufacturing (CAD/CAM) or 3D printing [11, 12]. However, digitalisation in dental education focused on practical training rather than theoretical learning content, which was principally imparted via face-to-face lectures. It can be hypothesised that without the COVID-19 pandemic, there was simply no need to transfer face-to-face lectures to digital learning formats. Online learning was not prescribed in dental education regulations in Germany. Meanwhile, this fact was altered in the new Dental Licensure Act, which officially became a force in the fall term of 2020. Currently, for the first time in history, dental education allows and claims digital learning formats as a supplement to face-to-face learning in Germany.

To achieve the highest possible educational success, new teaching concepts must be accepted by all participants [13]. Therefore, students’ and lecturers’ perceptions at the dental school of the Justus Liebig University Giessen (JLU, Germany) were evaluated after one semester of online learning at the end of the spring term of 2020 [9]. The results were predominantly positive in both groups, postulating an amount of online learning among the students of 53.2% (24.9) (mean (standard deviation)) and lecturers of 38.6% (21.5) beyond the COVID-19 pandemic.

Even though students and lecturers gained their first experience in online learning, the first wave of enthusiasm in the context of perpetuating dental education in spring term 2020 [9] might distort the perception of online learning. Although students considered online learning as a good option during the COVID-19 pandemic, they also felt that they were unprepared for the practical part of the curriculum with only online learning [9], demonstrating the importance of face-to-face teaching in dental education in the preclinical and clinical curricula. Furthermore, in the last two years, an increasing number of students have reported a lack of education due to distance learning. Therefore, it must be discussed whether online learning should be pursued without a valid reason if students do not feel adequately prepared to practice as dentists.

Despite these obstacles, online learning has also been shown to offer certain advantages in content delivery and in aspects of quality of life that allow more flexibility in daily planning and time management [14, 15].

Given the prevalence of practice-based teaching in dental education, there are concerns about the effectiveness and quality of online learning compared to established face-to-face teaching. Even in the absence of COVID-19, data on student and lecturer perceptions of online learning in dental education are scarce. Most studies only focused on one group at one point in time and did not critically question the fact that the implementation of online learning was a result of an emergency situation [16].

To the best of our knowledge, no study has investigated in a follow-up setting how the students’ and lecturers’ perceptions of online learning changed over time. The findings are essential for the development of future curricula to conduct contemporary, beneficial dental education while imparting knowledge.

Therefore, this cross-sectional study aimed to analyse students’ and lecturers’ perceptions towards online learning after a five-semester experience. Two online questionnaires (one for the students and one for the lecturers) were used.

## Methods

### Teaching method

Due to the COVID-19 pandemic preclinical (1st to 6th semester) and clinical (7th to 10th semester) students, as well as lecturers, used synchronous formats such as live online courses via an online videoconferencing system (Webex Meetings, Cisco Systems, Düsseldorf, Germany) and asynchronous formats such as recorded lectures and seminars deposited on online platforms for self-study of the JLU university (Knowledge-Based Medical Education (k-MED) and Stud.IP). Furthermore, a combination of synchronous and asynchronous formats (e.g. lectures and scripts on online platforms and ‘consultation hours’ for students’ questions) was offered. All online courses were led by lecturers, except for students presentations in the clinical semesters in the framework of patient case-based seminars.

In addition, technical checks were provided to familiarise students and lecturers with digital teaching systems. In the following semesters, an increasing amount of theoretical content was transferred back from online learning to face-to-face, so that all students could evaluate both online and face-to-face formats.

### Online survey

In cooperation with the Teaching Evaluation Service Centre of the JLU, two online questionnaires (one for students and one for lecturers) from 2020 [9] were adjusted and provided via the online survey tool LimeSurvey (Hamburg, Germany) (supplementary information). The survey contained evaluative statements on handling (the way students or lecturers dealt with online learning), didactic benefits (the way students or lecturers indented online learning as helpful regarding dental education), and motivation (the way students or lecturers were enthusiastic about online learning). Furthermore, participants were asked to state what made their daily lives easier and to make recommendations for personal benefits in the development of future curricula. Finally, questions regarding demographics were asked. The lecturers were additionally asked for their increased expertise in digital teaching formats.

A five-point Likert scale [17] was used in which all study participants could mark their responses as ‘agree or disagree’. Each question was open to non-response. The survey was evaluated anonymously and conducted in accordance with the ethical standards of the Institutional Review Board and local ethics committee of the JLU (Ref. No. 84/20).

Preclinical and clinical students participating in the dental curriculum of the JLU university in spring term 2022 (n = 291) and all lecturers (n = 56) with at least one year of teaching experience in face-to-face and online learning formats were invited via e-mail to participate in this study on 23 August 2022. Two reminders were sent after two and six weeks. The survey was closed on 17 October 2022. Only fully completed questionnaires were considered for the data evaluation.

Statistical analyses were performed using SPSS Statistics (version 28, IBM, Armonk, NY, USA). The distribution of the responses is presented as the mean and standard deviation. In addition to descriptive statistics, some aspects were further analysed, defining a level of significance of p < .05. First, differences between the study group and the basic group regarding gender and semester distribution were investigated using the chi-square test. Differences between asynchronous and synchronous online formats concerning the frequency of participation were statistically analysed using a sign test. The correlation between the following responses: ‘camera switched off during synchronous online learning’ and ‘I do not feel comfortable participating in online learning formats’ was further investigated using the Mann–Whitney test, and the correlation between ‘technical device’ and ‘reason for usage’ was analysed using the chi-squared test with Fisher’s exact test. The correlation between ‘preferred learning format’ and ‘teaching (face-to-face vs. online learning)’ as well as ‘preferred learning format’ and ‘easiness to learn with online learning’ were examined separately for students with and without patient contact using the Mann–Whitney test. Furthermore, a t-test was applied to analyse data regarding the amount of online learning in the future curriculum. Spearman’s correlation was applied to investigate the influence of teaching experience in lecturers’ group regarding the amount of online learning and the items in Tables 1, and pairwise comparisons were conducted using the Mann-Whitney test to analyse the suitability of the course type for future online learning between students and lecturers.

**Table 1:** Correlation between technical device and reason for usage classified to students’ and lecturer’s group

Group	Technical Device	Why did you choose this device? [N]				
		I find it most suitable.	It was just available.	It was the only device with camera and microphone.	I don’t know.	Total
Students	Laptop	98	14	10	2	124
	Tablet	25	4	2	0	31
	Desktop computer	10	0	2	0	12
	Smartphone	0	2	2	0	4
Lecturers	Laptop	9	3	4	0	16
	Tablet	1	0	0	0	1
	Desktop computer	12	5	1	0	18
	Smartphone	0	0	0	0	0

## Results

### Study group

A total of 174 students (105 women, 53 men, 1 inter/divers, 15 no answer) participated on this survey, with it being distributed to 95 students in preclinical education without patient contact (1st to 6th semester, mean age 24.4 ± 3.3 years) and 64 students in clinical education with patient contact (7th to 10th semester, mean age 25.8 ± 3.2 years). Fifteen students could not be classified into the preclinical or clinical curriculum due to the absence of answers in the questionnaire. Furthermore, 40 lecturers (19 women, 17 men, 4 no answer; teaching experience median 10 years, confidence interval 9.5–18.5 years) completed the survey. This represented a response rate of 59.8% for students and 71.4% for lecturers.

Within the lecturer group, no statistically significant differences between participants and basic groups with regard to gender were found (chi-squared test, p = .907), whereas fewer male students than females participated in this survey compared to the basic group (chi-squared test, p = .021). The semester distribution of the study group was representative of that of the basic group (chi-squared test, p = .948).

### Handling

The majority of lecturers stated that they conducted digital teaching formats such as synchronous online learning (n = 18), followed by a combination of synchronous and asynchronous formats (n = 8), and asynchronous online learning (n = 6), while information on eight lecturers was missing. Most students stated that they participated in both formats (synchronous and asynchronous) completely or in the majority of events (79.3% and 85.6%), no significant difference between the two formats regarding the frequency of participation could be detected (sign test, p = .546).

The majority of students stated they had a trouble-free workplace (88.5%) and adequate equipment (90.2%) with a stable Internet connection (90.8%) to participate in online learning. While 72.5% of all lecturers conducted online teaching from their workplace with LAN (n = 23), 97.1% of all students reported participating in online learning from home with WLAN (n = 158). Most students reported participating in online learning alone, and only one-third switched on their video cameras during synchronous online formats. However, no significant correlation between the two categories, ‘camera switched off during synchronous online learning’ and ‘I do not feel comfortable participating to online learning formats’ could be detected (Mann–Whitney test, p = .171).

The most used devices for online learning in the student group were laptops, followed by tablets, desktop computers, and smartphones, while lecturers mainly used their desktop computers or laptops, defining this as the most suitable device. Only one lecturer used the tablet. Table 1 displays the correlation between technical devices and the reason for usage. In the lecturer group, no statistically significant difference was observed (chi-squared test with Fisher’s exact test, p = .562); students exhibited a level of significance (chi-squared test with Fisher’s exact test, p = .010). The smartphone is conspicuous, which, despite the fact that it does not appear to be suitable, was used by four students.

Students with and without patient contact were asked about their preferred learning format (Table 2). In general, all formats were answered by at least 30% of both groups. However, more students of preclinical semesters (those without patient contact) stated to learn on screen rather by practicing and more likely by themselves instead of together with other students. In contrast, more clinical students with patient contact preferred learning by practicing with other students. A significant correlation between ‘preferred learning format’ and ‘teaching (face-to-face vs. online learning)’ was found only in students without patient contact for the following items: ‘on screen’, ‘by practicing’, and ‘with other students together’ (Table 2; Mann–Whitney test). In contrast, regarding the correlation between ‘preferred learning format’ and ‘easiness to learn with online learning’ in both groups, the significance of some items was archived (Table 2; Mann–Whitney test).

**Table 2:** Correlation between ‘preferred learning format’ and ‘teaching (face-to-face vs. online learning)’ as well as ‘preferred learning format’ and ‘easiness to learn with online learning’ classified for students without and with patient contact

Students without patient contact								Students with patient contact							
The theoretical teaching content is easy to learn with online learning.			In generally, I prefer face-to-face rather than online learning.			preferred learning format				In generally, I prefer face-to-face rather than online learning.			The theoretical teaching content is easy to learn with online learning.		
p-value*	N	Mean (SD)	p-value*	N	Mean (SD)					Mean (SD)	N	p-value*	Mean (SD)	N	p-value*
0.035	59	1.81 (0.86)	< 0.001	58	3.59 (1.19)	Yes	on screen (e.g. PC, laptop, tablet)		Yes	3.34 (1.37)	35	0.270	1.66 (0.80)	35	0.004
	31	2.32 (1.11)		31	2.16 (1.10)	No			No	2.97 (1.38)	29		2.38 (1.01)	29	
0.668	40	1.90 (0.84)	0.892	39	3.10 (1.25)	Yes	by listening (e.g. in lectures in presence or digitally)		Yes	3.16 (1.37)	31	0.934	2.03 (0.91)	31	0.542
	50	2.06 (1.08)		50	3.08 (1.41)	No			No	3.18 (1.40)	33		1.94 (1.03)	33	
0.022	65	2.11 (0.92)	0.003	64	2.83 (1.22)	Yes	by practicing (e.g. exercises on manikins or patients)		Yes	3.15 (1.38)	47	0.822	2.02 (0.92)	47	0.417
	25	1.68 (1.07)		25	3.76 (1.42)	No			No	3.24 (1.39)	17		1.88 (1.11)	17	
0.003	31	2.35 (0.84)	0.004	32	2.56 (1.05)	Yes	with other students together		Yes	2.96 (1.27)	24	0.297	2.25 (0.90)	24	0.047
	59	1.80 (1.00)		57	3.39 (1.40)	No			No	3.30 (1.44)	40		1.83 (0.98)	40	
0.660	52	1.96 (0.99)	0.265	50	3.22 (1.33)	Yes	by oneself		Yes	3.39 (1.45)	31	0.187	2.10 (1.14)	31	0.595
	38	2.03 (0.97)		39	2.92 (1.35)	No			No	2.97 (1.43)	33		1.88 (0.78)	33	
0.058	43	2.19 (0.98)	0.134	40	2.85 (1.21)	Yes	by reading (e.g. books)		Yes	2.97 (1.43)	30	0.283	2.27 (1.05)	30	0.039
	47	1.81 (0.95)		49	3.29 (1.41)	No			No	3.35 (1.32)	34		1.74 (0.83)	34	
0.004	44	2.25 (0.89)	0.546	43	3.00 (1.25)	Yes	by writing down what has previously been learned		Yes	3.28 (1.36)	29	0.586	2.17 (1.14)	29	0.276
	46	1.74 (1.00)		46	3.17 (1.42)	No			No	3.09 (1.40)	35		1.83 (0.79)	35	

M = mean, SD = standard deviation, N = number of valid answers. Type of answer: 1 = strongly agree, 5 = strongly disagree),*= Mann–Whitney test

Remarkably, neither students nor lecturers found it difficult to follow or create online learning content. The lecturers rated the aspects of concentration, tiredness, and daily planning differently. While students claimed that online instruction allowed them to focus better, this was not the case for lecturers. However, lecturers disagreed with getting tired of digital teaching, whereas students did. When asked about daily scheduling, the majority of the students indicated that they were able to plan their daily routine better as a result of online teaching, this was less true for the lecturers.

Table 3 displays the perceptions of the students and lecturers regarding the handling of online learning in detail.

**Table 3:** Comparison of students’ and lecturers perceptions regarding the handling of online learning

Students without patient contact		Students with patient contact		Item descriptions	Lecturers	
M (SD)	N	M (SD)	N		M (SD)	N
4.0 (0.94)	94	3.67 (1.05)	63	I found it difficult to follow the teaching content/ I found it difficult to create digital teaching formats.	4.53 (0.72)	32
n.a.	n.a.	n.a.	n.a.	The preparation effort is higher compared to face-to-face teaching.	3.27 (1.28)	33
2.95 (1.37)	84	3.35 (1.27)	62	In the context of online learning, I dare to ask questions more often than face-to-face./ In the context of online learning, students are more likely to dare to ask questions than in face-to-face teaching.	2.72 (1.09)	36
2.35 (1.06)	93	2.27 (1.04)	63	I had the opportunity to actively contribute to the teaching (e.g. questions or similar).	n.a.	n.a.
2.09 (1.07)	94	2.33 (1.15)	63	I can follow the course content with concentration/ I can concentrate better on my teaching.	2.72 (1.14)	32
2.68 (0.95)	94	2.65 (0.99)	63	I get tired of the digital teaching formats on the screen.	3.27 (1.38)	33
1.45 (0.78)	94	1.61 (0.76)	61	The digital implementation of the teaching allows me to plan my daily routine well./ The digital implementation of the teaching allows me to plan my daily routine well.	2.65 (1.25)	34

M = mean, SD = standard deviation, N = number of valid answers. Type of answer: 1 = strongly agree, 5 = strongly disagree)

### Didactic benefit and motivation

In general, the perceptions of students and lecturers differ regarding the didactic benefits of online learning. While students, regardless of patient contact, assessed the didactic benefit of online learning mostly as positive, lecturers reported a decrease in the quality and quantity of knowledge among students due to online teaching. Even though online teaching is a component of modern education, lecturers prefer face-to-face instruction when it comes to preparing students for practical courses. Furthermore, in terms of acceptance of online learning, lecturers indicated that they would prefer face-to-face learning to online learning, whereas students clearly preferred online teaching. However, both students and lecturers agreed that online learning formats were useful not only in the context of the COVID-19 pandemic but should also be used in the dental curriculum in the future. In the case of motivation, the opinion towards online learning was very balanced between students and lecturers with a positive attitude. Detailed information regarding perceptions of didactic benefits is displayed in Table 4.

**Table 4:** Comparison of students’ and lecturers’ perception regarding the didactic benefit and motivation of online learning

Students without patient contact		Students with patient contact		Item description	Lecturers	
M (SD)	N	M (SD)	N		M (SD)	N
2.21 (1.22)	47	2.95 (1.27)	60	The quality and quantity of knowledge has remained unchanged among students during online learning (compared to solely face-to-face teaching).	3.58 (1.23)	36
2.57 (1.19)	82	2.61 (1.19)	62	By participating on the online learning, I feel well prepared for the practical part of education./Preparing students for the practical courses works well with online learning formats.	3.11 (1.22)	37
1.99 (0.98)	90	1.98 (0.97)	64	The theoretical teaching content is easy to learn with online learning./ The theoretical teaching content is easy to teach with online learning.	2.38 (0.98)	37
3.09 (1.34)	89	3.17 (1.38)	64	In generally, I prefer face-to-face rather than online learning.	2.12 (0.84)	34
1.88 (1.07)	90	1.83 (0.97)	64	I think online learning formats belong to modern teaching in dentistry.	1.87 (0.78)	38
2.15 (0.97)	95	2.29 (1.07)	63	The use of new digital teaching methods (e.g. online teaching) motivates me to learn./ The use of new digital teaching methods (e.g. online teaching) motivates me to teach.	2.4 (1.0)	35
3.96 (1.08)	89	3.63 (1.26)	63	I do not feel comfortable participating to online learning formats because I miss the communication in person with the lecturers./ I do not feel comfortable teaching online learning formats because I miss the communication in person with students.	3.03 (1.45)	33
4.02 (1.18)	87	4.00 (1.22)	63	In the context of the COVID-19 pandemic, online learning formats were useful, but beyond the pandemic, they should not find further application in dental curriculum.	3.82 (1.27)	38
n.a.	n.a.	n.a.	n.a.	I perceived the students to be disciplined and attentive during the digital teaching formats.	2.84 (1.27)	31

M = mean, SD = standard deviation, N = number of valid answers. Type of answer: 1 = strongly agree, 5 = strongly disagree

**Table 5:** Comparison of descriptive statistics of students’ and lecturers perceptions regarding the overall assessment of online learning versus face-to-face learning

Students without patient contact		Students with patient contact		Item description	Lecturers	
M (SD)	N	M (SD)	N		M (SD)	N
2.91 (0.99)	92	2.62 (0.73)	63	Online learning Follow-up effort/ Preparation effort	2.25 (0.91)	32
2.11 (0.81)	92	2.54 (0.74)	63	Face-to-face learning Follow-up effort/ Preparation effort	2.59 (0.74)	34
2.10 (0.71)	92	2.38 (0.83)	63	Online learning Knowledge transfer	2.54 (0.82)	35
2.51 (0.83)	93	2.25 (0.78)	64	Face-to-face learning Knowledge transfer	2.00 (0.68)	36
2.52 (1.02)	92	2.58 (1.01)	64	Online learning Opportunities to ask questions	2.82 (0.87)	34
2.22 (1.06)	94	1.83 (0.87)	64	Face-to-face learning Opportunities to ask questions	1.59 (0.73)	37
2.60 (0.89)	88	2.76 (0.98)	63	Online learning Number of tips from lecturer	2.76 (0.96)	34
2.52 (1.04)	91	2.08 (0.88)	61	Face-to-face learning Number of tips from lecturer	1.86 (0.88)	35
2.43 (0.95)	92	2.55 (1.13)	64	Online learning Fun factor	2.88 (1.17)	33
2.39 (1.03)	93	2.16 (0.83)	62	Face-to-face learning Fun factor	1.74 (0.75)	34
3.82 (1.02)	93	3.65 (1.10)	63	Online learning Stress during the course	3.47 (0.83)	34
2.43 (1.04)	95	3.10 (0.98)	61	Face-to-face learning Stress during the course	3.19 (1.09)	36
2.99 (1.01)	86	3.11 (1.06)	62	Online learning Threshold of interaction	2.71 (1.06)	34
3.17 (1.10)	87	3.40 (1.06)	62	Face-to-face learning Threshold of interaction	3.53 (1.40)	36

M = mean, SD = standard deviation, N = number of valid answers (total: N=).Type of answer: 1 = very high, 5 = very low

A high level of agreement was found between students and lecturers regarding the aspects of better feasibility, easier interaction in terms of the ability to ask questions and receive tips from lecturers, as well as less stress. However, students without patients showed a somewhat lower agreement. Nevertheless, the students stated that they enjoyed online learning more than the lecturers did. The lecturers were of a different opinion on this point; moreover, fewer tips from lecturers and queries were possible (Table 5). A significant correlation between ‘teaching experience’ within the lecturers’ group and the items in Table 5 could not be identified (Spearman’s correlation, p > .05).

### Increase in expertise

Even if the COVID-19 pandemic had not occurred, 75% of the lecturers stated that they would have dealt with the topic of ‘digital teaching formats’ to the same extent (n = 16) or not in that intensity (n = 14). Only 17.5% reported that they would have ’not at all’ focused on online learning without COVID-19. Regarding the attitude towards the topic of online learning formats and the motivation to implement online learning formats, a positive change was observed.

**Fig. 1:**
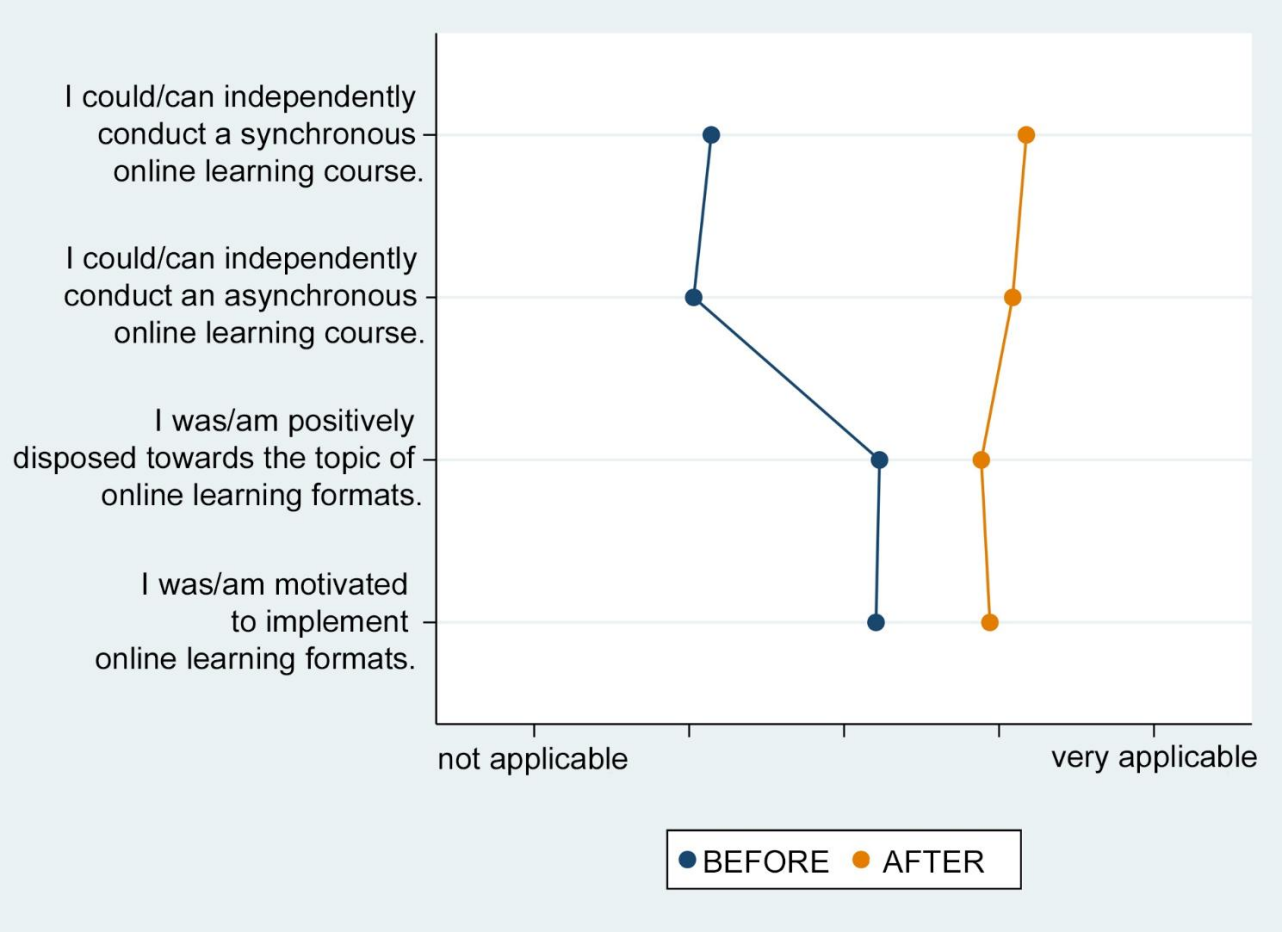
Self-evaluation of change in the expertise of lecturers before and after five semesters of experience in online learning implemented into the dental curriculum

In terms of competency growth in online teaching compared to before the COVID-19 pandemic (before the spring term of 2020) and after five semesters with online learning formats, there was an obvious increase in expertise among lecturers. Regarding knowledge about online learning formats, a clear increase was also observed, and the implementation of synchronous and asynchronous online learning courses was indicated as significantly improved (Fig. 1).

### Prospects for future curriculum beyond COVID-19 pandemic

Most students stated that a combination of synchronous and asynchronous formats (n = 93) would be suitable for future dental curricula, followed by asynchronous (n = 43) and synchronous (n = 21) formats, whereas 12 students preferred face-to-face teaching. Lectures also see a combination of synchronous and asynchronous formats (n = 17) as valuable but are followed by synchronous (n = 14) and asynchronous (n = 6) formats. Three lecturers did not recommend maintaining online teaching. The answers of five students were missing.

Regarding the optimal amount of online learning on theoretical teaching, students demanded with 49.5% (mean) (standard deviation: 25.1) a significantly higher percentage of online learning compared to lecturers 34.1% (standard deviation: 24.1) (t-test, p = .01). However, no significant difference was observed between the students with and without patient contact regarding the optimal amount of online learning (t-test, p = .775). A significant correlation within the lecturers’ group between ‘teaching experience’ and the ‘amount of online learning’ could not be identified (Spearman’s correlation, p = .380).

**Fig. 2:**
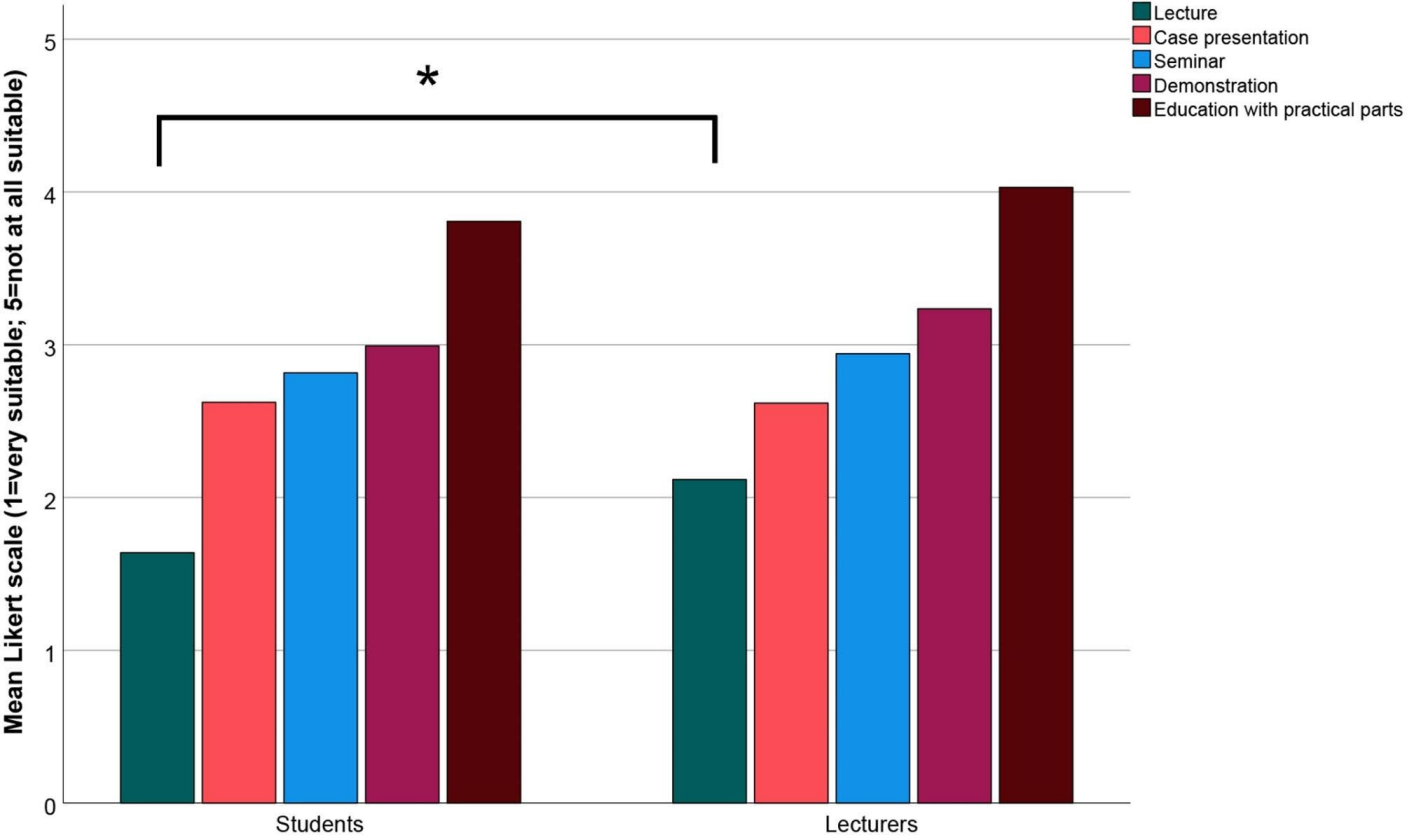
Suitability of different teaching formats evaluated by students and lecturers for future online learning in the dental curriculum

In contrast to the amount of online learning, students and lecturers agreed on the suitability of course type for future online learning. Both rated lectures as the most suitable teaching format, followed by case presentations, seminars, and demonstrations. However, education with a practical aspect and patients were considered unsuitable for online learning (Fig. 2). A significant difference was only observed between students and lecturers regarding the item ‘lecture’ (Mann–Whitney test, p < .001).

## Discussion

Dental education has changed dramatically in the wake of the COVID-19 pandemic worldwide [18, 19]. Online formats have been rapidly and innovatively used to support teaching and learning, which was previously non existent in dental education in Germany because patient education requires hands-on practice, and the dental curriculum was designed in its original format as a full-time, face-to-face format [20]. The availability of online teaching in education has significantly increased [21, 22]. The COVID-19 pandemic has resulted in a paradigm shift in dental education and future clinical provision [23]. All lecturers and students had to adapt to the new concept because of the emergency situation.

In general, lecturers have a positive attitude towards the increased use of online teaching. Although over 60% of lecturers at our dental school had no experience with online teaching before the COVID-19 pandemic, they adapted quickly to online learning, as already observed [24], and the knowledge gained regarding the implementation of online learning was very high. As described in previous studies, the lecturers interviewed wanted to continue teaching online even after the pandemic [9]. It was noticeable that with regard to the equipment they used to participate in the online course, the students mainly did not use their smartphones, although it can be assumed that they would always have had this at hand, instead of laptops, tablets, or desktop computers. This shows that they were mostly well equipped to participate in the online course.

The data show that students prefer online teaching in dental education more than lecturers, which is in line with other authors [25, 26]. Moreover, students in preclinical semesters without patient contact were more likely to learn on screen, whereas more students in clinical programs with patient contact preferred to learn by practicing with other students. One explanation for this could be that preclinical semesters, due to their lack of know-how of patient contact teaching and missing related teaching together, may not have experienced this learning as positive. However, it can be discussed if student led seminars or journal clubs might increase the attractiveness of online courses within clinical students due to the possibility to actively participate on the lesson design.

The results of this study clearly highlight how university dental education has changed since the pandemic. There is a new work-life reality. On the one hand, this educational change has several advantages. Students’ emphasize these in terms of improved work-life balance. Students may feel less stressed as a result of their improved ability to plan daily schedules because of online learning. Because our findings show that only one student attended online instruction away from home, this most likely fits better into their daily schedule. Presumably, online learning has implications for daily routine, which students indicated was easier with respect to online teaching. This better time flexibility for students has already been described in another study [24]. Generally, clinical dental work outside of teaching does not occur at home. Therefore, many lecturers have been affiliated with dental clinics. This constraint could be suspected as the reason why only four lecturers reported teaching from home. This was already indicated by a prior investigation in 2020, which showed a large proportion (62.9%) of lecturers conducted lectures from dental clinics [9].

However, the new integrated online approach may have negative implications for students’ and lecturers’ well-being. For example, it has to be discussed that the increasing number of online sessions can lead to increased stress, e-mails, and the demand for ‘constant presence’ may cause problems. Spending hours in front of electronic devices can affect mental health [27]. However, there are significant challenges that must be addressed if online teaching in dental education is to be effective and beneficial to both students and lecturers. However, the positively evaluated aspects of online learning, such as increased student motivation, easier participation, and reduced time commitment, can be used to improve future dental curricula.

The future of dental education is uncertain after the end of the pandemic, but new opportunities for online teaching pose a challenge because decision-making for future teaching and learning may be evaluated differently than it was before the pandemic [28]. More than 75% of the lecturers stated that they would not have dealt with online teaching to the same extent without the COVID-19 pandemic, and 17.5% stated that they would not have dealt with the topic of online learning at all without the COVID-19 pandemic situation. Furthermore, in terms of attitudes and motivation to use online learning formats, as well as the increase in competence in online teaching compared to before the COVID-19 pandemic (before the spring term of 2020), there was a distinct increase in competence among lecturers. The importance of practical training was again emphasised. It should also be pointed out here that physical training through mental training can bring about similar improvements in fine practical skills as practical training [29, 30], underlining mental training should also play a role in dental education and should also be considered in future teaching with regard to practical skills development. However, more preclinical semester students reported learning alone than with other students and online, as mentioned above. Clinical students with patients, on the other hand, preferred learning by practicing together with other students, which underlines the need for practical education. Dental schools cannot extensively adopt online teaching practices This aspect is also highlighted in other studies as a disadvantage of online teaching [31–33].

A limitation of this study is that the data collection was completed within seven weeks and queried for a five-semester period. This may have influenced our results. In particular, a retrospective survey of this long period may not be as accurate. Therefore, when interpreting these results, one should be aware that this was a retrospective study. Due to the single center design of this study, it should be considered that the results cannot be representative for all universities, especially for universities already using online teaching in education before COVID-19. Thus, further multicenter study investigating these aspects would be desirable.

As expected, the response rate resulted in a smaller sample size compared with a prior study by 2020[9]. However, as the survey was anonymous, we were unable to identify those who did not respond.

With regard to future curriculum development, almost all students consider a combination of synchronous and asynchronous formats to be useful, similar to the majority of lecturers. In this context, students would like to see a higher proportion of online learning than lecturers, which could be due to their improved daily routine. The fact that lecturers teach from dental clinics does not represent an advantage for them in their daily routines.

While lectures are considered best suited for online teaching by both students and lecturers, it is evident that online teaching cannot replace practical training, which underlines the importance of practical training in dental teaching in the future.

## Conclusions

The COVID-19 pandemic has transformed the dental education field. The data of this study confirm the results of a study conducted in 2020[9], which indicates that online learning was not only a good solution to dental education during the pandemic, but was requested by students and lecturers even without an ‘emergency situation’. We concluded that both dental students and lecturers are ready to support online learning. In this regard, the combination of face-to-face and online courses seems to be a future trend in dental education, as practical courses in dental education should continue to be based on face-to-face formats. Therefore, in terms of planning a future dental curriculum, it is important to think thoroughly about which activities can take advantage of digital tools. Thus, it can be concluded that despite the enormous acceptance of online teaching by students and lecturers, traditional practical dental education should remain a cornerstone of dental education.

### Electronic supplementary material

Below is the link to the electronic supplementary material.

Supplementary Material 1

## Data Availability

The datasets analysed during the current study are not publicly available due to university requirements but are available from the corresponding author on reasonable request.

## Abbreviations

3D: Three dimensions
CAD/CAM: Computer-aided design/ computer-aided manufacturing
COVID-19: Coronavirus disease 2019
k-MED: Knowledge-based Medical Education
LAN: Local area network
SARS-CoV-2: Severe acute respiratory syndrome coronavirus 2
WLAN: Wireless local area network
