# Supplementary material for: Update of students’ and lecturers’ perspectives on online learning in dental education after a five-semester experience due to the SARS-CoV-2 (COVID-19) pandemic: insights for future curriculum reform
Source: BMC Med Educ. 2023 Aug 8;23:556. doi: 10.1186/s12909-023-04544-2 (PMC10411012; doi:10.1186/s12909-023-04544-2)
Supplement: Supplementary file 1 — Supplementary Material 1 [file 12909_2023_4544_MOESM1_ESM.pdf]

Questionnaire Students

Handling

1. How often did you participate on the synchronous digital teaching formats offered?

Please select one of the following answers:

- not at all
- in minority
- nearly half
- in majority
- completely
- no answer

2. How often did you participate on the asynchronous digital teaching formats offered (e.g. recorded lectures and seminars deposited on k-med)?

- not at all
- in minority
- nearly half
- in majority
- completely
- no answer

3. Which device did you mainly use for the participation on the online learning courses?

Please select only one of the following answers:

- Smartphone
- Tablet
- Laptop
- PC
- No answer

4. Why did you choose this device?

Please select only one of the following answers:

- It was just available.
- It was the only device with camera and microphone.
- I find it most suitable.
- I don't know.

5. Please share your perspective regarding future dental curriculum. Which digital teaching formats would be suitable for online learning in the new dental licensure act?

Please choose one of the following answers:

- Synchronous formats such as live online seminars/ lectures (e.g. Webex).
- Asynchronous formats such as recorded lectures and seminars deposited on k-med on online platforms for self-study (e.g. k-MED, Stud-IP).
- Combination of synchronous and asynchronous formats (e.g. lectures and scripts on online platforms and "consultation hours" for students' questions).
- None at all. Dentistry should only be taught as a face-to-face course.

6. To what extent do the following statements apply to online learning?

Please select the appropriate answer for each point:

	strongly disagree	disagree	neutral	agree	strongly agree	no answer
I found it difficult to follow the teaching content.	○	○	○	○	○	○
In the context of online learning, I dare to ask questions more often than face-to-face.	○	○	○	○	○	○
I had the opportunity to actively contribute to the teaching (e.g. questions or similar).	○	○	○	○	○	○
I can follow the course content with concentration.	○	○	○	○	○	○
I get tired of the digital teaching formats on the screen.	○	○	○	○	○	○
The digital implementation of the teaching allows me to plan my daily routine well.	○	○	○	○	○	○

Didactic benefit and motivation

7. To what extent do the following statements apply to the online learning?

Please select the appropriate answer for each point:

	strongly disagree	disagree	neutral	agree	strongly agree	no answer
	○	○	○	○	○	○
The quality and quantity of knowledge has remained unchanged during the digital teaching formats (compared to solely face-to-face teaching).	○	○	○	○	○	○
By participating on the online learning, I feel well prepared for the practical part of education.	○	○	○	○	○	○
The theoretical teaching content is easy to learn with online learning.	○	○	○	○	○	○
I generally prefer face-to-face rather than online learning.	○	○	○	○	○	○

I think online learning formats belong to modern teaching in dentistry.	○	○	○	○	○	○
The use of new digital teaching methods (e.g. online teaching) motivates me to learn.	○	○	○	○	○	○
I do not feel comfortable participating to online learning formats because I miss the communication in person with the lecturers.	○	○	○	○	○	○
In the context of the COVID-19 pandemic, online learning formats were useful, but beyond the pandemic, they should not find further application in dental curriculum.	○	○	○	○	○	○

8. How do you prefer to learn?

Please select all that apply:

- by oneself
- with other students together
- on screen (e.g. PC, laptop, tablet)
- by practicing (e.g. exercises on manikins or patients)
- by listening (e.g. in lectures in presence or digitally)
- by reading (e.g. books)
- by writing down what has previously been learned

Overall assessment

9. Please assess face-to-face learning regarding the following aspects:

Please select the appropriate answer for each point:

	very low	low	medium	high	very high	no answer
Follow-up effort	○	○	○	○	○	○
Knowledge transfer	○	○	○	○	○	○
Opportunities to ask questions	○	○	○	○	○	○
Number of tips from lecturers	○	○	○	○	○	○
Fun factor	○	○	○	○	○	○
Stress during the course	○	○	○	○	○	○

	very low	low	medium	high	very high	no answer
Threshold of interaction with teachers (from "very low" = high interaction to "very high" = no interaction)	○	○	○	○	○	○

10. Please assess online learning regarding the following aspects:

Please select the appropriate answer for each point:

	very low	low	medium	high	very high	no answer
Follow-up effort	○	○	○	○	○	○
Knowledge transfer	○	○	○	○	○	○
Opportunities to ask questions	○	○	○	○	○	○
Number of tips from lecturers	○	○	○	○	○	○
Fun factor	○	○	○	○	○	○
Stress during the course	○	○	○	○	○	○
Threshold of interaction with teachers (from "very low" = high interaction to "very high" = no interaction)	○	○	○	○	○	○

11. How large should be the amount of online learning regarding the theoretical part of education (independent of COVID-19) in the future curriculum?

Please enter your answer here:

____ Percent (%)

Numbers in percent from 0 to 100

12. Please assess for which type of course you think digital teaching formats are suitable:

	not at all suitable	rather not suitable	neutral	rather suitable	very suitable	no answer
Seminar	○	○	○	○	○	○
Lecture	○	○	○	○	○	○
Demonstration	○	○	○	○	○	○

	not at all suitable	rather not suitable	neutral	rather suitable	very suitable	no answer
Case presentation	○	○	○	○	○	○
Education with practical parts and patients	○	○	○	○	○	○

Final questions

13. What is your gender?

Please select only one of the following answers:

- male
- female
- inter / diverse
- no answer

14. Please state your age in years.

Please enter your answer here: ___ years

15. In which semester are you currently studying?

Please enter your answer here:

____ semester

16. How did you predominantly participate in the online learning?

Please select only one of the following answers:

- alone
- physically together with other students
- other

17. Did you predominantly participate in the digital teaching formats with the video image switched on?

Please select only one of the following answers:

- Yes
- No

18. What type of internet connection did you use mostly?

Please select only one of the following answers:

- Mobile network
- WLAN

- LAN
- No answer

19. Do you have a workplace that allows you to participate in online learning without disruptions?

Please select only one of the following answers:

- Yes
- No

20. Do you have adequate equipment that enables you to participate in online learning without interference (camera, headset)?

Please select only one of the following answers:

- Yes
- No

21. How often did you have problems with your internet connection so that you could not follow relevant content properly?

Please select only one of the following answers:

- never
- among the minority of events
- about half of the events
- for the majority of events
- always
- no answer

22. Where did you spend most of your time during online learning?

Please select only one of the following answers:

- at home
- at the dental clinic
- at the workplace of the (part-time) job
- in public (e.g. in transport, café/restaurant etc.)
- Other

<u>Questionnaire Lecturers</u>

Handling

1. Which online learning format(s) have you predominantly offered for your courses since the spring term 2020 (COVID-19 pandemic)?

Please select only one of the following answers:

- Synchronous formats such as live online seminars/ lectures (e.g. Webex).

- Asynchronous formats such as recorded lectures and seminars deposited on k-med on online platforms for self-study (e.g. k-MED, Stud-IP).
- Combination of synchronous and asynchronous formats (e.g. lectures and scripts on online platforms and "office hours" for questions).
- None at all, since all teaching formats are face-to-face.
- Other

2. Which device did you mainly use for the online learning courses?

Please select only one of the following answers:

- Smartphone
- Tablet
- Laptop
- PC
- No answer

3. Why did you choose this device?

Please select only one of the following answers:

- It was just available.
- It was the only device with camera and microphone.
- I find it most suitable.
- I don't know.

4. Please share your perspective regarding future dental curriculum. Which digital teaching formats would be suitable for online learning in the new dental licensure act?

Please choose one of the following answers:

- Synchronous formats such as live online seminars/ lectures (e.g. Webex).
- Asynchronous formats such as recorded lectures and seminars deposited on k-med on online platforms for self-study (e.g. k-MED, Stud-IP).
- Combination of synchronous and asynchronous formats (e.g. lectures and scripts on online platforms and "office hours" for questions).
- None at all. Dentistry should only be taught as a face-to-face course.

5. To what extent do the following statements apply to online learning?

Please select the appropriate answer for each point:

	strongly disagree	disagree	neutral	agree	strongly agree	no answer
I found it difficult to create digital teaching formats.	○	○	○	○	○	○
The preparation effort is higher compared to face-to-face teaching.	○	○	○	○	○	○

	strongly disagree	disagree	neutral	agree	strongly agree	no answer
In the context of online learning, students are more likely to dare to ask questions than in face-to-face teaching.						
I can concentrate better on my teaching.						
I get tired of the digital teaching formats on the screen.						
The digital implementation of the teaching allows me to plan my daily routine well.						

Didactic benefit and motivation

6. To what extent do the following statements apply to the online learning?

Please select the appropriate answer for each point:

	strongly disagree	disagree	neutral	agree	strongly agree	no answer
The quality and quantity of knowledge has remained unchanged among students during online learning (compared to solely face-to-face teaching).						
Preparing students for the practical courses works well with online learning formats.						
The theoretical teaching content is easy to teach with online learning.						
I generally prefer face-to-face teaching rather than online learning for my teaching.						
I think online learning formats belong to modern teaching in dentistry.						
The use of new digital teaching methods (e.g. online teaching) motivates me to teach.						
I do not feel comfortable teaching online learning formats because I						

miss the communication in person with students.						
In the context of the COVID-19 pandemic, online learning formats were useful, but beyond the pandemic, they should not find further application in dental curriculum.	○	○	○	○	○	○
I perceived the students to be disciplined and attentive during the digital teaching formats.	○	○	○	○	○	○

7. Would you have dealt intensively with the topic of "digital teaching formats" even without the Covid-19 pandemic?

Please select only one of the following answers:

- No, absolutely not.
- I can't judge.
- Yes, but not in intensity.
- Yes, to the same extent as necessitated by the pandemic.

Overall assessment

8. Please assess face-to-face learning regarding the following aspects:

Please select the appropriate answer for each point:

	very low	low	medium	high	very high	no answer
Preparation effort	○	○	○	○	○	○
Knowledge transfer	○	○	○	○	○	○
Possibility to ask questions	○	○	○	○	○	○
Number of tips for students	○	○	○	○	○	○
Fun factor	○	○	○	○	○	○
Stress during the course	○	○	○	○	○	○
Threshold of interaction with students (from "very low" = high interaction to "very high" = no interaction)	○	○	○	○	○	○

9. Please assess online learning regarding the following aspects:

Please select the appropriate answer for each point:

	very low	low	medium	high	very high	no answer
Preparation effort	◯	◯	◯	◯	◯	◯
Knowledge transfer	◯	◯	◯	◯	◯	◯
Possibility to ask questions	◯	◯	◯	◯	◯	◯
Number of tips for students	◯	◯	◯	◯	◯	◯
Fun factor	◯	◯	◯	◯	◯	◯
Stress during the course	◯	◯	◯	◯	◯	◯
Threshold of interaction with students (from "very low" = high interaction to "very high" = no interaction)	◯	◯	◯	◯	◯	◯

10. How large should be the amount of online learning regarding the theoretical part of education (independent of COVID-19) in the future curriculum?

Please enter your answer here:

____ Percent (%)

Numbers in percent from 0 to 100

11. Please assess for which type of course you think digital teaching formats are suitable:

	not at all suitable	rather not suitable	neutral	rather suitable	very suitable	no answer
Seminar	◯	◯	◯	◯	◯	◯
Lecture	◯	◯	◯	◯	◯	◯
Demonstration	◯	◯	◯	◯	◯	◯
Case presentation	◯	◯	◯	◯	◯	◯
Education with practical parts and patients	◯	◯	◯	◯	◯	◯

Increase in expertise

12. To what extent do the following statements about digital teaching formats apply to you?

Please select the applicable answer for each item:

	This is how it was BEFORE the Covid-19 pandemic (before SoSe 2020) without online learning formats ...					So it is AFTER five semesters with online learning formats ...				
	not applicable	rather not applicable	neutral	rather applicable	very applicable	not applicable	rather not applicable	neutral	rather applicable	very applicable
I could/can independently conduct a synchronous online learning course.	○	○	○	○	○	○	○	○	○	○
I could/can independently conduct an asynchronous online learning course.	○	○	○	○	○	○	○	○	○	○
I was/am positively disposed towards the topic of online learning formats.	○	○	○	○	○	○	○	○	○	○
I was/am motivated to implement online learning formats.	○	○	○	○	○	○	○	○	○	○

Final questions

13. What is your gender?

Please select only one of the following answers:

- ○ male
- ○ female
- ○ inter / diverse
- ○ no answer

14. How many years of active teaching experience do you have (excluding parental leave, sabaticcal, extended illness)?

Please enter your answer here: ___ years

15. What type of internet connection did you use mostly?

Please select only one of the following answers:

- o Mobile network
- o WLAN
- o LAN
- o No answer

16. How often did you have problems with your internet connection during online learning courses?

Please select only one of the following answers:

- o never
- o among the minority of events
- o about half of the events
- o for the majority of events
- o always
- o no answer

17. Where did you spend most of your time during online learning?

Please select only one of the following answers:

- o at home (in home office)
- o own office at dental clinic
- o common room at dental clinic
- o Other
